# Dietary *Arthrospira platensis* in Rainbow Trout (*Oncorhynchus mykiss*): A Means to Reduce Threats Caused by CdCl_2_ Exposure?

**DOI:** 10.3390/toxics10120731

**Published:** 2022-11-26

**Authors:** Mahdi Banaee, Federica Impellitteri, Hamid Evaz-Zadeh Samani, Giuseppe Piccione, Caterina Faggio

**Affiliations:** 1 Aquaculture Department, Faculty of Natural Resources and the Environment, Behbahan Khatam Alanbia University of Technology, Behbahan 47189, Iran; 2 Department of Veterinary Sciences, Polo Universitario dell’Annunziata, University of Messina, 98168 Messina, Italy; 3 Department of Chemical, Biological, Pharmaceutical and Environmental Sciences, University of Messina, Viale Ferdinando Stagno, d’Alcontres 31, 98166 Messina, Italy

**Keywords:** dietary supplement, CdCl_2_, toxicity, oxidative stress, serum parameters, *Arthrospira platensis*

## Abstract

The rainbow trout (*Oncorhynchus mykiss*) is one of the most commercially sought-after freshwater fish species and one of the most farmed in the world. On the other hand, aquaculture breeding frequently results in outbreaks of infectious diseases and pests, and compromises the production and welfare of fish. *Arthrospira platensis* (known as “Spirulina”) has been used as a supplement in diets to enhance fish welfare in recent years because of its beneficial properties. This study aimed to assess the possible protective effects of *Arthrospira platensis* on rainbow trout specimens exposed to three different doses of the toxicant CdCl_2_. The experiment was carried out using five experimental treatments of 40 individuals each: control group; group II (0.2 mg CdCl_2_ per kg of commercial fish feed); group III (0.2 mg Kg^−1^ of CdCl_2_ plus 2.5 g per kg of *A. platensis*); group IV (0.2 mg Kg^−1^ of CdCl_2_ plus 5 g per kg of *A. platensis*); group V (0.2 mg Kg^−1^ of CdCl_2_ plus 10 g per kg of *A. platensis*). During the experiment, dietary supplementation of *A. platensis* normalized all serum and blood parameters altered by the presence of CdCl_2_. *A. platensis* also had a protective effect on markers of oxidative stress.

## 1. Introduction

One of the freshwater fish species that is most commercially in demand and one of the most widely farmed worldwide is the rainbow trout (*Oncorhynchus mykiss*). The global production of rainbow trout in 2016 was roughly 0.8 million tonnes, accounting for 2% of all fish aquaculture worldwide production [1,2,3]. However, intensive production systems are susceptible to infectious diseases and pests, which generate stressful conditions in fish and interfere with their well-being.

The application of immunostimulants through food inclusion has been suggested as a possible approach to improving health and preventing illness [4]; it is also of great relevance for maintaining the quality of the fish and increasing their yield [5]. Numerous immunostimulants, including vitamins, chitin, glucans, microbes, and other readily accessible by-products, have been shown to have favorable effects [6,7]. The fact that medicinal plants and their derivatives include a variety of active ingredients makes them suitable as supplements to the nutritional diets of aquatic organisms such as rainbow trout. Indeed, these substances often result in beneficial effects on such organisms. [7,8,9]. Numerous recent studies indicate the benefits of these natural product constituents on the health of numerous aquatic animals. These compounds appear to be able to directly interact with the immune response [10,11,12] by boosting defensive activity, and they also have an antioxidant impact, protecting animals from oxidative stress [13,14]. Among these beneficial and natural sources of immunostimulants, there is *Arthrospira platensis*, a blue-green microalga (cyanobacteria) that has been utilized for generations as a food source. It has a spiral structure that varies in number and narrowness and is typically 0.1 mm in diameter [15]. The United Nations World Food Conference has put algae on its list of healthy foods due to its nutritional properties. *Arthrospira platensis* is thought to contain antiviral, antibacterial, antioxidant, anti-diabetic, anti-cancer, and anti-inflammatory properties in addition to its nutritional properties. As a result, this microalga is regarded as a superfood [16] and seems to also be effective in fish farming, since it appears to boost fish development [17], stress tolerance, and resistance to hunger [18,19]. It can also positively influence intestinal flora, lipid digestion, and coloration [20]. An interesting study [21] shows that in some species such as *Oreochromis niloticus*, the use of feed consisting of these algae improved antioxidant biomarkers, particularly in the gills and liver. Due to its positive contributions and in particular its protein component, *A. platensis* has been adopted as a supplement within diets in recent years [22]. For example, it is also used to reduce oxidative damage [23].

Indeed, fish in aquaculture facilities are exposed to many stressors daily (crowding stress, handling stress, pathogens, parasites, etc.), all of which affect fish health, growth, and performance. Heavy metals are the biggest threat to the health of aquatic organisms. Metals usually penetrate the surface and underground waters through industrial sewage contamination, mining activities, and municipal and agricultural wastewater. Cadmium (Cd) is one of the most known transition metals and is widely used in the electronics, plastics, and battery industries [24]. Therefore, Cd can be found in various aquatic ecosystems due to direct industrial discharges into waters or watersheds. Much of the cadmium that reaches aquatic systems tends to accumulate in sediments, and under certain conditions, re-enters the water column. Thus, finfish and shellfish may be exposed to water-borne Cd through feed or water. Although fish can eliminate a significant amount of Cd from their bodies, exposure to Cd may lead to biochemical, physiological, and behavioral disorders in these animals [25,26,27].

In this study, a stress condition was simulated by exposing rainbow trout to CdCl_2_, a known toxicant resulting from anthropogenic pollution and in particular from industry [28,29,30]. Furthermore, due to its poor excretion, Cd is toxic even at low doses [31,32] and has a negative impact on several metabolic processes in fish tissues and organs, and it has therefore been highly studied in several model organisms [33] and fish species [34,35,36,37,38]. It was then decided to evaluate whether the inclusion of *A. platensis* in the diet could contribute to increasing the robustness of the fish and alleviate the negative effects of stress. For this reason, this study aimed to assess the protective effects of *A. platensis* on rainbow trout specimens subjected to three different CdCl_2_ concentrations. Using *A. platensis,* it might be possible to reduce the side-effects of Cd in aquatic animals by increasing the efficiency of the detoxification system.

## 2. Materials and Methods

### 2.1. Sampling of the Specimens

The present study was conducted at the Fish Farm, Almas-Dime Village, Koohrang, Charmahal and Bakhtiari Province, in Iran, during the period from April to July 2016. According to the National Ethical Framework for Animal Research in Iran, two hundred animals belonging to the rainbow trout (*Oncorhynchus mykiss*) species were used for the performed analyses. Each of the specimens weighed 200 ± 10 g.

### 2.2. Experimental Design and Diet

The experiment lasted for 21 days, during which a total of 200 juvenile rainbow trout (*Oncorhynchus mykiss*) were randomly distributed in 5 concrete gullies (10,000 L) and acclimatized in aerated freshwater (16 ± 2 °C; pH, 7.4 ± 0.2; 100% water exchange/day; a natural photoperiod) for a fortnight prior to the experiment. During the acclimatization period, the fish were fed twice daily with commercial feed from the “Faradaneh Company, Sharkourd”, produced in Iran (Table 1). Moreover, CdCl_2_ (99% purity, Merck Co., Darmstadt, Germany) was orally administered to fish and the *Arthrospira platensis* treatment. Dry *A. platensis* powder was obtained from Sinamicroalgae Co. (Qeshm, Iran).

The experiment was carried out using a completely randomized model with five experimental treatments of 40 individuals each:

- Group I, or the control group, was fed on a basal diet without any treatment;
- Group II was fed with a basal diet, and 0.2 mg CdCl_2_ per kg of commercial fish feed;
- Group III was fed simultaneously with 0.2 mg Kg^−1^ of CdCl_2_ and supplemented with 2.5 g *A. platensis*;
- Group IV was fed simultaneously with 0.2 mg Kg^−1^ CdCl_2_ and supplemented with 5 g *A. platensis* per kg commercial fish feed;
- Group V was fed simultaneously with 0.2 mg Kg^−1^ CdCl_2_ and supplemented with 10 g *A. platensis* per kg commercial fish feed.

According to the information provided on brochure of commercial diet purchased from Faradaneh Co. (Sharkourd, Iran), the proximate analysis of basal diet indicated 39–40% crude protein, 10.5–11% crude lipid, and 2–2.5% fiber [39].

### 2.3. Preparing Samples

Twelve fish from each group were collected after 21 days of CdCl_2_ exposure and *Arthrospira platensis* (*AP*) treatment and anaesthetized with clove powder (150 mg L^−1^). Next, blood samples were collected from the caudal vein with a 2.5 cc syringe and poured into a microtube (2 mL). After centrifuging the blood samples (4 °C, 15 min, 6000 rpm), the supernatant was separated to measure blood biochemical parameters using biochemical reagents obtained from ParsAzmun Co., Iran (Baharstan Industrial Township, Karaj, Alborz, Iran). Then, the fish were euthanized, and their livers were extracted, rinsed with a physiological solution, and homogenized in a cold phosphate buffer solution for 2 min (pH: 4.7). The resulting homogenized solution was centrifuged at 150,000 rpm for 15 min at 4 °C. The supernatant was collected for biochemical parameter measurements and kept at −70 °C until biochemical analysis [40].

### 2.4. Blood Biochemical Parameter Analyses Performed

Relevant blood parameters were evaluated, such as the glucose, cholesterol, triglyceride, total protein, albumin, creatinine contents and aspartate aminotransferase, alanine aminotransferase, alkaline phosphatase, lactate dehydrogenase, creatinine phosphokinase, gamma-glutamyltransferase, and butyrylcholinesterase activities. These parameters were measured using biochemical reagents purchased from ParsAzmun Co. (A producer of clinical chemistry and immunoturbidimetric reagents, in Iran). All serum parameters were determined strictly according to the manufacturer’s instructions. Briefly, biochemical reagents that included buffer and substrate or enzyme were added to a certain amount of sample and then incubated. Finally, changes in wavelength absorption were measured with a spectrophotometer, and concentrations of analytes or activities of enzymes were calculated following the manufacturer’s instructions.

Glucose contents in serum were determined using glucose oxidase [41]. Triglyceride and cholesterol concentrations were assessed in the presence of lipase and cholesterol esterase, respectively [42]. Pyric acid was used to measure creatinine [43]. Total protein and albumin were estimated using bromocresol green and cupric ion, respectively. Globulin content was also calculated by subtracting albumin from total protein [44].

The level of the aspartate aminotransferase (AST) enzyme was determined using a paired reaction with malate dehydrogenase in the presence of NADH. The enzyme can react with alanine and a-ketoglutarate to produce glutamate and pyruvate during the alanine aminotransferase (ALT) assay. The AST and ALT activities were determined by measuring the absorbance variation in 3 min at 340 nm. The lactate dehydrogenase (LDH) activity was evaluated by measuring the conversion of pyruvate in L-lactate and monitoring NADH oxidation for 3 min at 340 nm. The activity of alkaline phosphatase (ALP) was determined by measuring the conversion of p-nitrophenol phosphate to nitrophenol at 405 nm in an alkaline buffer. CPK (creatinin phosphokinase) activity was measured using creatinine phosphate and adenosine diphosphate (ADP) as substrates at 340 nm [45]. BChE activity was determined using butyrylcholine at 405 nm [46]. GGT activity was evaluated using glutamic acid at 405 nm [45]. Total immunoglobulin (Ig) levels were detected using polyethylene glycol following the technique described by Banaee et al. [47], at 540 nm.

Using the FRAP reagent, total antioxidant capacity was determined based on the plasma’s ferric-reductive ability. The freshly made FRAP reagent consisted of 5 mL of 10 mmol/L TPTZ (2,4,6-tripyridyl-s-triazine) solution in 40 mmol/L HCl, 5 mL of 20 mmol/L FeCl_3_, and 50 mL of acetate buffer (0.3 mol/L, pH = 3.6). Following that, 3 mL of FRAP reagent was combined with 100 µL of supernatant aliquots. As the conversion rate of the ferric tri-pyridyl-s-triazine complex (Fe^3+^-TPTZ) to ferrous tri-pyridyl-s-triazine complex (Fe^2+^-TPTZ) at pH 3.6 and 25 °C is directly proportional to the total antioxidant concentration in the sample, through this method it was possible to obtain the data. The Fe^2+^-TPTZ complex has a strong blue color that may be detected using a UV/VIS spectrophotometer for 5 min at 593 nm. Furthermore, calculations were performed using a calibration curve of FeSO_4_-7H_2_O (100 to 1000 µM/L) [48].

Malondialdehyde (MDA) level was expressed as mol/g tissue and was estimated by using the modulated thiobarbituric acid test. In this context, 500 µL of supernatant was mixed with 2500 µL of trichloroacetic acid (20%) and 1000 µL of thiobarbituric acid (67%), in a Pyrex tube. Tubes were then placed in hot water at 100 °C for 15 min. After boiling, the organic phase of the chromogenic substrate was extracted with 1000 µL of distilled water and 5000 µL of n-butanol:pyridine (15:1). The mixture was subsequently centrifuged at 2000 rpm for 15 min at 4 °C. The reaction resulted in a pink-colored complex that was measured using a spectrophotometer at 532 nm to detect MDA levels, and its concentration was quantified using the MDA standard, which was prepared and synthesized from tetraethoxypropane and absolute ethanol [49].

Catalase (CAT) activity was determined by making some variations to the above-mentioned kits. Indeed, the hydrogen peroxidase assay was used, which is based on the formation of a stable complex by the addition of ammonium molybdate. The reaction produced a yellow complex, whose concentration was measured at 405 nm [50].

$$\mathrm{Catalase}\;\mathrm{activity}\;\left({\mathrm{kU}\cdot L}^{-1}\right)=\frac{A\left(sample\right)-A\;\left(blank1\right)}{A\left(blank2\right)-A\left(blank3\right)}\times 271$$

*Blank1* included 1.0 mL substrate, 1.0 mL molybdate, and 0.2 mL distilled water; *blank2* included 1.0 mL substrate, 1.0 mL molybdate, and 0.2 mL buffer; and *blank3* included 1.0 mL buffer, 1.0 mL molybdate, and 0.2 mL buffer.

Superoxide dismutase, glutathione reductase, glutathione peroxidase, and glucose 6 phosphate dehydrogenase activities were tested in supernatant obtained from liver tissue homogenate using biochemical reagents purchased from Biorex-Fars Co. (Shiraz, Iran). SOD activity was assayed using xanthine oxidase and xanthine to produce superoxide anions. GPx activity was estimated utilizing reduced glutathione (GSH) and cumene hydroperoxide. GR activity was detected using NADPH and oxidized glutathione (GSSG) [51]. G6PDH activity was measured using glucose 6-phosphate as substrate [52].

All biochemical endpoints above-mentioned were measured using a UV/VIS spectrophotometer (Biochrom Libra S22 model, Waterbeach Cambridge, CB25 9PE, UK)

### 2.5. Measurement of Cadmium Bioaccumulation

After the autopsy, muscle, skin, gills, and liver samples were cut and dried in the oven. Then, 1 g of each tissue was mixed with 5 mL of H_2_O_2_ and kept overnight at room temperature (25 °C). Next, samples were blended with 15 mL of acid mixture concentrated HNO_3_/HCl (3:1) and digested at 150 °C for 12 h. Digested samples were cooled and filtered using a Whatman filter (0.22 μm) and diluted in deionized water to a final volume of 25 mL. Finally, cadmium concentrations in the samples were estimated using inductively coupled plasma optical emission spectrometry (ICP–OES spectrometer provided by SPECTRO Analytical Instruments GmbH Boschstr. 10, 47533 Kleve, Germany) [53].

### 2.6. Data Analyses

To verify the normality of data, the Kolmogorov–Smirnov normality test was performed using SPSS, version 22 (Chicago, IL, USA). One-way ANOVA was used to analyze the data. The significant differences between experimental groups were calculated with Tukey’s post hoc test at both *p* < 0.05 and *p* < 0.01.

## 3. Results

### 3.1. Comments on the Clinical Status

Neither mortality nor clinical indications were documented in either the control or treatment groups during the experiment. In the CdCl_2_ exposed group, however, only fast opercular movement was seen, indicating increased respiration.

### 3.2. Serum Biochemical Parameters

Regarding all test statistics, degrees of freedom, and *p*-values (*p* < 0.01) and (*p* < 0.05) of all ANOVAs conducted, please refer to the tables in the Supplementary Materials.

After exposure to CdCl_2_, the amount of total protein decreased compared to the control; the difference was significant (*p* < 0.01) for the first group. In contrast, all groups that received the *AP* supplement had a progressive increase in the total amount of protein compared to the control group, as opposed to exposure to Cd alone. As for albumin levels, these remained superimposable to those of the control group (Table 2).

The values of globulins decreased following exposure to cadmium (*p* < 0.05); treatment with *AP*, especially in the group exposed to the highest *AP* concentrations, brought the values back to a level similar to that of the control. In this case, the values were not significantly different. Total immunoglobulin levels significantly (*p* < 0.01) increased in fish exposed to 0.2 mg CdCl_2_, relative to controls. In contradistinction, the use of CdCl_2_ feed was responsible for significant (*p* < 0.01) increases in certain blood values—in particular, glucose, cholesterol, and triglycerides. Again, the administration of *AP* resulted in an average drop in values in the groups with *AP* concentrations of 2.5 g (group III) and 5.0 g (group IV). The use of a greater dose of 10.0 mg in the final experimental group resulted in a decrease in the values compared to the control (Table 2).

The creatinine level in group I (0.2 mg CdCl_2_ alone) was significantly (*p* < 0.01) higher in comparison to the control group. Experimental groups treated with increasing concentrations of *AP* showed a trend directly proportional to the administered *AP* concentrations. Indeed, fish belonging to group III, treated with 2.5 g of *AP*, showed significant divergence in creatinine level compared to the control group, despite the amount of creatinine administered being less than in group II. Fish belonging to groups IV and V treated with 5 and 10 g of *AP* showed creatinine levels comparable to that of the control group (Table 2).

In fish fed with 0.2 mg CdCl_2_ (II group), AST activity in serum was significantly (*p* < 0.01) higher compared to the control group (I group). The AST activity in fish treated with the highest concentration of *AP* was similar to the AST activity in the control group. In the groups treated with 2.5 g (group III) and 5 g (group IV) of *AP*, the divergence with the control group was still higher, while at the highest administered concentration of *AP* (V group), AST activity decreased compared to the control group, despite the value is not statistically significant (Table 3).

Fish which were CdCl_2_-treated, belonging to group II (0.2 mg CdCl_2_), showed a significant (*p* < 0.01) increase in ALT activity in serum. Group III fish, treated with 2.5 g SP, showed a significant decreasing trend in ALT activity. In groups IV (5 g *AP*) and V (10.0 g *AP*), ALT values in comparison to the control group were maintained (Table 3).

CdCl_2_ treatment of group-II fish (0.2 mg CdCl_2_ alone) resulted in a relevant significant (*p* < 0.01) increase in LDH activity compared to the control group. The group-III fish, treated with 2.5 g of *AP*, also showed a trend of LDH activity significantly higher than the control. The 5 g *AP*-treated group (group III) had less high LDH activity than group II but still higher than the control group. A non-significant (*p* > 0.05) LDH activity difference with respect to the control group was highlighted in the group (V group) treated with 10 g of *AP* (Table 3).

The results revealed that the activity of ALP significantly (*p* < 0.01) increased in the serum of fish orally exposed to 0.2 mg CdCl_2_ without *AP*. In comparison, no significant changes were observed between fish treated with 0.2 mg CdCl_2_ plus *AP* (5 and 10 mg *AP* per kg feed) and the control group.

GGT activity in the sera of group II (0.2 mg CdCl_2_ without *AP*) was significantly (*p* < 0.01) higher compared to the control group. The same was found for the 2.5 g *AP*-treated group (group III), and the trend in GGT activity was decreased in the experimental group treated with 10 g *AP* (group V) compared to the control, though the difference was non-significant (Table 3).

In CdCl_2_-treated fish (group II—0.2 mg CdCl_2_ without *AP*), enzymatic BchE activity in serum showed a significant decrease compared to the control group. In groups treated with various concentrations of *AP*, BchE activity was significantly (*p* < 0.01) decreased compared to the control group, and the trend was directly proportional to the *AP* concentration administrated (Table 3).

Group II (0.2 mg CdCl_2_ alone) showed a significant (*p* < 0.01) increase in CPK activity in serum, whereas the groups treated with higher concentrations of *AP* (III, IV, and V groups) maintained comparable enzymatic CPK activities compared to the control group (Table 3).

### 3.3. Tissue Antioxidant and Oxidative Stress Markers

**Figure 1:**
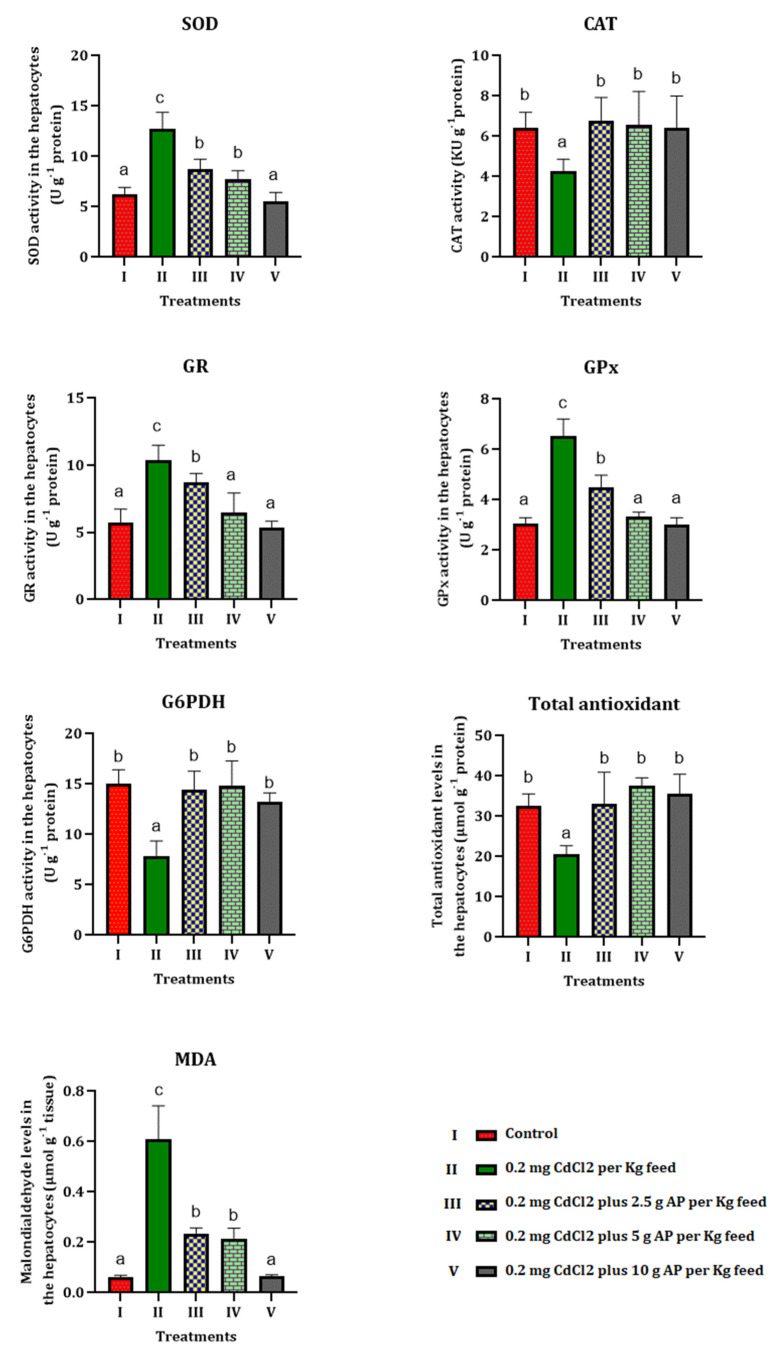
Results on tissue antioxidant values and oxidative stress markers. Different letters show significant changes in values (*p* < 0.05) and (*p* < 0.01), and the same letter shows there was no significant difference between the experimental groups. One-way ANOVA was used to analyze the data. Duncan’s test was used for the comparison, with confidence levels of 95% (*p* = 0.05) and 99% (*p* = 0.01).

Tissue homogenate of the livers of *Oncorhynchus mykiss* was prepared to investigate the antioxidant status and oxidative stress markers in the CdCl_2_-treated group of fish and the preventive role of *AP* (Figure 1).

The level of total cellular antioxidants in the homogenized liver tissue of fish exposed to CdCl_2_ decreased significantly (*p* < 0.01) compared to the control. The administration of 2.5, 5, and 10 g of *AP*, in contrast, led to an increase in the total antioxidant level, which recovered to the value of the control in group V (basal diet with 0.2 mg CdCl_2_, plus 10 g *AP*).

The MDA levels in the livers of the fish treated with CdCl_2_ alone (group II) were significantly (*p* < 0.01) higher compared to the control group; and the gradual inclusion of *AP* in the diet of the fish (2.5, 5.0, 10.0 g *AP*) had a beneficial effect, bringing the MDA values back to the same level as the control group, thereby eliminating the alteration produced by CdCl_2_. A significant (*p* < 0.05) decrease in CAT values was found in group II (0.2 mg CdCl_2_ without *AP*), which started to gradually increase with the addition of *AP* in groups III (2.5 g *AP*), IV (5 g *AP*) and V (10.0 g *AP*), reaching, in the group with the highest *AP* concentration, the control group’s values. In the group exposed to CdCl_2_ alone, a significant (*p* < 0.01) increase in hepatic SOD was observed, which gradually subsided with *AP* supplementation of 2.5, 5, and 10.0 g. Dietary supplementation of *AP* resulted in almost full recovery and normalization of hepatic SOD values (Figure 1).

Glutathione reductase (GR) levels also increased significantly (*p* < 0.01) in group II, and then decreased with increasing *AP* supplementation in the fish’s diet. Similar results were observed in the analyses of the hepatic glutathione peroxidase (GPx), which was significantly (*p* < 0.01) elevated in the group exposed to CdCl_2_ alone, and then gradually decreased to levels overlapping those of the control in the group fed the highest concentration of *AP* (group V). Glucose-6-phosphate dehydrogenase values (*p* < 0.01) decreased in the presence of the pollutant alone (group II) and then returned to the initial values in groups III (2.5 g *AP*), IV (5 g *AP*), and V (10.0 g *AP*), i.e., those in which *AP* was present in the diet (Figure 1).

### 3.4. Bioaccumulation of Cadmium

**Figure 2:**
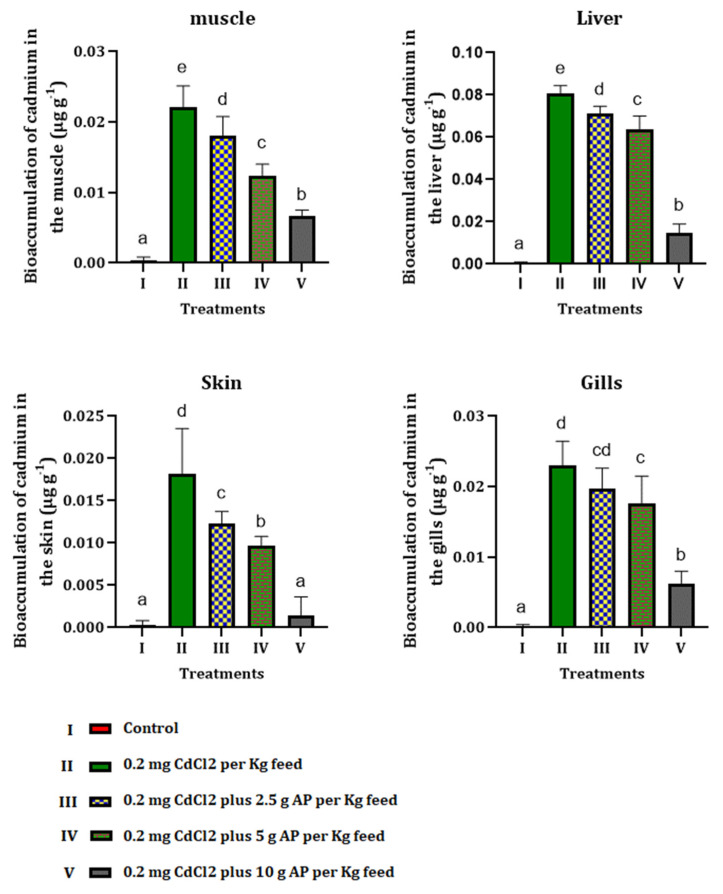
Bioaccumulation of cadmium in different tissues of fish orally exposed to CdCl_2_ and *Arthrospira platensis*. Different letters show significant changes in values (*p* < 0.05) and (*p* < 0.01), and the same letter by different numbers shows there was no significant difference between the experimental groups.

Results showed that feeding fish with a polluted diet by Cd increased its bioaccumulation levels in various tissues. Although administration of *AP* could mitigate Cd bioaccumulation, there was a significant difference between Cd contents in the different tissues of the experimental fish and control group (Figure 2).

## 4. Discussion

Cadmium is one of the most toxic heavy metals. It has a wide distribution in the environment. It is a non-essential heavy metal that can bioaccumulate and be toxic to organisms even at small concentrations [54]. It has also been demonstrated to induce free-radical formation, resulting in oxidative damage to lipids, proteins, and DNA [55,56]. The toxic effects of Cd are numerous, including stunted development and growth [57], disturbances in liver function [58], and pathological alterations in certain tissues and organs [59]. A 2018 study [60] assessed the acute sensitivity of the larval stage of *Nothobranchius furzeri* to cadmium in combination with a 4 °C temperature increase. Cadmium was found to be highly toxic, with 100% mortality being achieved very rapidly at the two highest concentrations. In addition, a recent study [61] showed that exposure to cadmium can also affect the maturation time and reproductive performance of fish. In this study, cadmium delayed maturation in females (*Nothobranchius furzeri*) and reduced adult mass and fecundity.

Furthermore, as the metal concentration rises and exceeds the capacity of the organisms’ detoxification mechanisms, a variety of deleterious consequences and increased mortality emerge [62,63]. The various reactions to Cd exposure are undoubtedly connected to oxidative stress, which is characterized as an imbalance between oxidant fluxes and antioxidant defenses [64], which in turn determines an increase in the activities of antioxidant enzymes. These findings are also in agreement with data obtained by Al-Asgah et al. in 2015 [65].

*Arthrospira platensis* is described as a powerful tonic for the immune system and for boosting animals’ growth and development. Due to these properties, *Arthrospira* is utilized as a supplemental ingredient in fish feeds, and increasingly, as a protein and vitamin supplement in aquarium feeds [16].

Interestingly, CdCl_2_ reduced the concentration of serum protein in exposed fish—in particular, total protein and globulins. As for albumin levels, these remained superimposable with those of the control group. Serum-globulin deficiency obviously suggests liver failure. Furthermore, it is suggested that the decrease in serum protein content is due to an increase in stressor levels in exposed fish [66,67]. Dietary supplementation of *AP* alleviates the toxicity induced by CdCl_2_, and the concentration of serum protein is restored to its normal level, reestablishing normal hepatic function.

During cadmium exposure to the rainbow trout specimens in the study, serum glucose and cholesterol were significantly elevated in the group exposed to CdCl_2_. This increase is a clear response to the damage that cadmium can cause in fish organs and is considered an indicator of acute stress due to the action of the hormone cortisol, which stimulates increases in glycogenesis and gluconeogenesis. Furthermore, a rise in serum cholesterol is thought to be a sign of cell-structure degradation in the membranes of kidney and muscle cells [68]. Thus, increases in cholesterol levels are good indicators of environmental stress in fish [69]. Serum cholesterol and glucose levels reverted to normal in the *AP*-treated groups, confirming *Arthrospira*’s anti-stress action against xenobiotic toxicity and its protective impact on liver tissue [58].

Triglycerides have the main function of supplying energy to cells and can be used as indicators of nutritional status. The present study showed a slight increase in serum triglyceride concentration in the cadmium-exposed group, which returned to the control level after *AP* treatment. This could be related to the liver failure of the fish [16]. Triglyceride levels in metal exposed *Perca flavescens* [70] were also altered in the same way. Furthermore, rises in triglyceride and cholesterol levels in fish plasma may be a physiological response to supply enough energy to mitigate the harmful effects of this contaminant [71].

AST and ALT are non-organ-specific enzymes and are found in two different cytosolic and mitochondrial isoforms in all animal tissues. The activity of AST and ALT enzymes in the blood can be used as an indicator of stress [65]. Increased AST and ALT values in fish reveal the exportation of enzymes from the liver to the bloodstream [72], which may indicate hepatocellular, mitochondrial, or cell membrane damage. AST plays a crucial role in both glutathione biosynthesis and gluconeogenesis in hepatocytes. Therefore, an increase in plasma AST activity may be indicative of oxidative stress and tissue damage in rainbow trout specimens after CdCl_2_ exposure. These findings agree with those of Shalaby et al. [71] from a study conducted in 2007, in which they found that sub-lethal concentrations of Cd caused significant increases in AST and ALT in *O. niloticus*. There is a similar result also in a study from 2015 [65]. In this research, dietary supplementation with *AP* improved liver function by reducing the activity of both hepatic transaminases and alkaline phosphatase. The antioxidant compounds in *AP*, including ß-carotene, vitamins, and minerals, are thought to contribute significantly to protecting hepatic tissues from xenobiotic damage. Indeed, *Arthrospira platensis* showed a hepatoprotective effect in CdCl_2_-exposed fish by dramatically lowering serum transaminase and ALP activities.

Regarding LDH values in fish exposed to cadmium, there was a highly significant increase compared to the control group. LDH plays an essential role in the conversion of lactate to pyruvate, NAD+ to NADH, and vice versa in both cases. Hypoxic conditions, dysfunction of mitochondrial oxidation, necrosis, or cell death can increase LDH activity in the blood [73,74,75]. Similar results were observed in the blood of carp (*Cyprinus carpio*) exposed to microplastics and cadmium in a 2019 study [47]. The influence of the *Arthrospira* treatment, on the other hand, helped to bring the values back to normal, again confirming its protective and beneficial effect.

An increase in GGT activity was also identified during the investigation. GGT is a membrane-bound enzyme involved in glutathione reformation and biodegradation and xenobiotic detoxification. Therefore, GGT activity is essential to provide amino acids for the synthesis and renewal of intracellular glutathione [76]. Increased GGT activity indicates depletion of cellular glutathione, especially in hepatocytes, resulting in oxidative stress [77].

BChE is found in all tissues and cells, including erythrocytes [78]. It is involved in, among other things, the transmission of cellular signals. Oxidative stress and lipid peroxidation could be responsible for a significant decrease in BChE activity [77] in fish exposed to CdCl_2_. In any case, the *Arthrospira*-treated groups appear to restore normal values, acting in opposition to oxidative stress.

CPK is present in high concentrations in animal muscle cells, heart tissue, gills, kidneys, and the brain and can be released into circulation because of cell injury [79]. Increased CPK activity in the serum of fish can be due to muscle or renal damage.

An increase in creatinine levels was also found in the cadmium-treated fish compared to the control fish. Creatinine is metabolically produced by the breakdown of creatine phosphate during muscle and protein metabolism. The kidneys take care of its excretion. Therefore, if the kidneys are not functioning properly, or have any injuries, the creatinine level in the blood increases. In fact, the blood creatinine level is an excellent biomarker for assessing the glomerular filtration rate [47]. By reversing CPK and creatinine elevation, *Arthrospira platensis* could play an important role in the prevention and treatment of liver and kidney diseases, especially those mediated by oxidative stress.

The SOD-CAT system is a key component of the antioxidant defense that can exert a protective effect against oxidative stress by converting hydrogen peroxide into oxygen and water [80]. During the experiment, there was a decrease in CAT activity, which may be due to the direct effect of the metal [81]. In general, the inhibition of CAT activity is related to the binding of metal ions to the enzyme’s -SH groups, which increases the H_2_O_2_ or superoxide radical concentrations [82]. The decrease in CAT can also be linked to overproduction of ROS or altered gene expression. Similar results were also obtained in other ecotoxicology studies on fish [83,84]. The high activity of SOD resulting from exposure to CdCl_2_ may indicate high production of superoxide anion radicals [85,86].

The potential of oxidative stress formation increases as overall antioxidant capability decreases [87,88,89,90]. Total antioxidant levels were shown to be lower in fish exposed to cadmium. The decrease in total antioxidants, along with the rise in MDA, implies that CdCl_2_ exposure may lead to oxidative stress in fish. MDA levels rose considerably after metal exposure compared to the control group. Peroxidation of essential macromolecules such as lipids and proteins can result in the formation of metabolites such as MDA and protein carbonyl derivatives [88,89,90]. As a result, elevated MDA can be a good biomarker for detecting oxidative stress and the failure of the antioxidant defense mechanism.

Results showed a significant increase in the GPx activity of hepatocytes after exposure to cadmium. This increase in GPx activity may hasten the conversion of H_2_O_2_ and other proxide radicals to H_2_O and O_2_ and may minimize the formation of ROS in tissues [77,88].

As G6PD is a regulatory enzyme for NADPH-dependent biotransformation and defense against oxidative stress, a reduction in G6PD activity may lead to a decrease in NADPH production. NADPH is essential to maintaining glutathione in the reduced form, which reduces peroxides and protects cells from oxidative damage in the course [89,90].

A significant decrease in the Cd bioaccumulation may be related to phytochemical compounds of *AP*, especially flavonoids, which can inhibit the absorption of heavy metals in the digestive system. Panche et al. [91] showed that flavonoids could decrease the metal accumulation rate in the biological system. Moreover, *AP* antioxidants can also play an essential role in the cadmium detoxification system and remove it from the fish’s body. Bhattacharya [92] found that the administration of *A. platensis* can alleviate the toxicity effects of heavy metals through increased cellular antioxidant capacity.

During the experiment, the role of *Arthrospira platensis* became evident in bringing all values altered by the presence of CdCl_2_ back to normal, compared to the control. In the case of oxidative stress markers, therefore, one can see how *A. platensis* exerted a protective effect.

## 5. Conclusions

According to the experiment conducted on *Oncorhynchus mykiss*, exposure to CdCl_2_ induced an alteration in serum biochemical parameters, alterations in liver function biochemical parameters, reductions in antioxidant enzyme activities, and increases in markers of oxidative stress. The study found that *Arthrospira platensis* supplementation, because of its beneficial multi-properties, provided nearly total protection, minimizing or eliminating the detrimental effects caused by the heavy metal utilized in the study. Additionally, these results suggest that inclusion of *Arthrospira platensis* into the diet of farmed fishes may help to boost their robustness to stress, which may positively affect the wellbeing, quality, and yield of fish in aquacultural production systems.

## Figures and Tables

**Table 1:** A proximate composition of the experimental diets for the rainbow trout.

Crude Protein	Control/ Group I	Group II: 0.2 mg Kg^−1^ CdCl_2_	Group III: 0.2 mg Kg^−1^ CdCl_2_ 2.5 g Kg^−1^ *AP*	Group IV: 0.2 mg Kg^−1^ CdCl_2_ 5.0 g Kg^−1^ *AP*	Group V: 0.2 mg Kg^−1^ CdCl_2_ 10.0 g Kg^−1^ *AP*
Dry matter %	90	90	90.52	90.75	91.2
Metabolizable energy (Kcal/g)	2.68	2.68	2.79	2.83	2.91
Crude protein %	40.33	40.33	41.09	41.83	42.63
Crude lipid %	11	11	11.09	11.18	11.27
Crude fiber %	2	2	2.26	2.51	2.76

**Table 2:** Results regarding analysis of serum biochemical parameters.

Serum Biochemical Parameters	Control/ Group I	Group II: 0.2 mg Kg^−1^ CdCl_2_	Group III: 0.2 mg Kg^−1^ CdCl_2_ + 2.5 g Kg^−1^ *AP*	Group IV: 0.2 mg Kg^−1^ CdCl_2_ + 5.0 g Kg^−1^ *AP*	Group V: 0.2 mg Kg^−1^ CdCl_2_ + 10.0 g Kg^−1^ *AP*
Total protein (g dL^−1^)	4.4 ± 0.4 ^b,c^	3.7 ± 0.3 ^a^	4.0 ± 0.3 ^a,b^	4.5 ± 0.5 ^c^	4.6 ± 0.4 ^c^
Albumin (g dL^−1^)	2.9 ± 0.2 ^a^	2.9 ± 0.3 ^a^	2.9 ± 0.4 ^a^	3.0 ± 0.3 ^a^	3.0 ± 0.4 ^a^
Globulins (g dL^−1^)	1.5 ± 0.4 ^b^	0.8 ± 0.3 ^a^	1.1 ± 0.6 ^a^	1.5 ± 0.4 ^b^	1.6 ± 0.6 ^b^
Total immunoglobulins (g dL^−1^)	1.4 ± 0.2 ^b^	0.9 ± 0.3 ^a^	1.3 ± 0.2 ^b^	1.2 ± 0.2 ^b^	1.4 ± 0.2 ^b^
Glucose (mg dL^−1^)	61.8 ± 9.7 ^a^	110.1 ± 10.5 ^c^	99.7 ± 5.7 ^b^	66.9 ± 11.2 ^a^	69.1 ± 9.8 ^a^
Cholesterol (mg dL^−1^)	204.2 ± 14.5 ^a^	281.6 ± 19.1 ^b^	237.1 ± 7.9 ^b^	270.5 ± 9.9 ^b^	204.9 ± 42.3 ^a^
Triglycerides (mg dL^−1^)	172.1 ± 29.3 ^a^	223.9 ± 23.8 ^b^	226.6 ± 12.2 ^b^	172.7 ± 27.3 ^a^	170.8 ± 27.7 ^a^
Creatinine (mg dL^−1^)	0.5 ± 0.3 ^a^	1.7 ± 0.2 ^c^	1.0 ± 0.2 ^b^	0.4 ± 0.0 ^a^	0.7 ± 0.1 ^a^

Different letters show significant changes in values (*p* < 0.05) and (*p* < 0.01), and the same letter shows there was no significant difference between the experimental groups.

**Table 3:** Results on serum biochemical parameters.

Serum Biochemical Parameters	Control/ Group I	Group II: 0.2 mg Kg^−1^ CdCl_2_	Group III: 0.2 mg Kg^−1^ CdCl_2_ + 2.5 g Kg^−1^ *AP*	Group IV: 0.2 mg Kg^−1^ CdCl_2_ + 5.0 g Kg^−1^ *AP*	Group V: 0.2 mg Kg^−1^ CdCl_2_ + 10.0 g Kg^−1^ *AP*
AST (U·L^−1^)	117.8 ± 9.7 ^a,b^	177.3 ± 60.7 ^c^	141.1 ± 18.2 ^b^	141.1 ± 20.5 ^b^	108.3 ± 5.0 ^a^
ALT (U·L^−1^)	13.1 ± 1.1 ^a^	27.3 ± 0.9 ^c^	19.1 ± 1.0 ^b^	12.5 ± 0.6 ^a^	12.9 ± 1.1 ^a^
ALP (U·L^−1^)	132.2 ± 12.6 ^a^	302.1 ± 55.8 ^c^	192.8 ± 42.5 ^b^	150.5 ± 22.0 ^a,b^	132.4 ± 17.8 ^a^
LDH (U·L^−1^)	386.5 ± 21.2 ^a^	743.8 ± 16.7 ^d^	580.8 ± 13.3 ^c^	428.5 ± 17.8 ^b^	363.9 ± 16.4 ^a^
GGT (U·L^−1^)	33.6 ± 3.2 ^a,b^	47.1 ± 7.9 ^c^	39.1 ± 5.5 ^b^	26.4 ± 5.1 ^a^	29.4 ± 3.7 ^a^
BchE (U·L^−1^)	1158.9 ± 45.2 ^d^	586.5 ± 91.3 ^a^	788.5 ± 52.6 ^b^	971.3 ± 45.5 ^c^	1023.6 ± 72.5 ^c^
CPK (U·L^−1^)	567.0 ± 89.3 ^a^	774.2 ± 152.8 ^b^	657.3 ± 119.7 ^a^	561.6 ± 64.2 ^a^	572.2 ± 64 ^a^

Different letters show significant changes in values (*p* < 0.05) and (*p* < 0.01), and the same letter shows there was no significant difference between the experimental groups.

## Data Availability

The datasets generated during and/or analyzed during the current study are available from the corresponding author on reasonable request.

## Supplementary Materials

The following supporting information can be downloaded at: https://www.mdpi.com/article/10.3390/toxics10120731/s1. Table S1: One-way ANOVA test of serum biochemical parameters—part a. Table S2: One-way ANOVA test of serum biochemical parameters—part b. Table S3: One-way ANOVA test of tissue antioxidant values and oxidative stress markers.
