# Supplementary material for: Dietary Arthrospira platensis in Rainbow Trout (Oncorhynchus mykiss): A Means to Reduce Threats Caused by CdCl2 Exposure?
Source: Toxics. 2022 Nov 26;10(12):731. doi: 10.3390/toxics10120731 (PMC9781257; doi:10.3390/toxics10120731)
Supplement: Supplementary file 1 [file toxics-10-00731-s001.zip › toxics-2038904-supplementary.pdf]

**Table S1:** One-way ANOVA test of serum biochemical parameters - part a.

**ANOVA**

| | | Sum of Squares | df | Mean Square | F | Sig. |
|---|---|---|---|---|---|---|
| AST | Between Groups | 25652.954 | 4 | 6413.239 | 7.042 | .000 |
| | Within Groups | 36429.263 | 40 | 910.732 | | |
| | Total | 62082.218 | 44 | | | |
| ALT | Between Groups | 1460.944 | 4 | 365.236 | 390.552 | .000 |
| | Within Groups | 37.407 | 40 | .935 | | |
| | Total | 1498.351 | 44 | | | |
| ALP | Between Groups | 184186.101 | 4 | 46046.525 | 39.153 | .000 |
| | Within Groups | 47042.365 | 40 | 1176.059 | | |
| | Total | 231228.466 | 44 | | | |
| LDH | Between Groups | 922300.235 | 4 | 230575.059 | 773.853 | .000 |
| | Within Groups | 11918.283 | 40 | 297.957 | | |
| | Total | 934218.518 | 44 | | | |
| GGT | Between Groups | 2435.970 | 4 | 608.993 | 21.374 | .000 |
| | Within Groups | 1139.668 | 40 | 28.492 | | |
| | Total | 3575.638 | 44 | | | |
| CPK | Between Groups | 301235.508 | 4 | 75308.877 | 6.986 | .000 |
| | Within Groups | 431175.160 | 40 | 10779.379 | | |
| | Total | 732410.668 | 44 | | | |
| BChE | Between Groups | 1781305.437 | 4 | 445326.359 | 108.767 | .000 |
| | Within Groups | 163772.811 | 40 | 4094.320 | | |
| | Total | 1945078.248 | 44 | | | |
| AChE | Between Groups | 3235279.800 | 4 | 808819.950 | 28.188 | .000 |
| | Within Groups | 1147733.258 | 40 | 28693.331 | | |
| | Total | 4383013.057 | 44 | | | |

**Table S2:** One-way ANOVA test of serum biochemical parameters - part b.

**ANOVA**

| | | Sum of Squares | df | Mean Square | F | Sig. |
|---|---|---|---|---|---|---|
| Protein | Between Groups | 5.150 | 4 | 1.287 | 8.534 | .000 |
| | Within Groups | 6.034 | 40 | .151 | | |
| | Total | 11.184 | 44 | | | |
| Albumin | Between Groups | 3.909 | 4 | .977 | 10.915 | .000 |
| | Within Groups | 3.581 | 40 | .090 | | |
| | Total | 7.490 | 44 | | | |
| Golbulins | Between Groups | 4.796 | 4 | 1.199 | 5.706 | .001 |
| | Within Groups | 8.406 | 40 | .210 | | |
| | Total | 13.202 | 44 | | | |
| Ig | Between Groups | 1.861 | 4 | .465 | 8.396 | .000 |
| | Within Groups | 2.217 | 40 | .055 | | |
| | Total | 4.078 | 44 | | | |
| Glocose | Between Groups | 17132.021 | 4 | 4283.005 | 46.827 | .000 |
| | Within Groups | 3658.564 | 40 | 91.464 | | |
| | Total | 20790.585 | 44 | | | |
| Creatinine | Between Groups | 10.267 | 4 | 2.567 | 87.352 | .000 |
| | Within Groups | 1.175 | 40 | .029 | | |
| | Total | 11.443 | 44 | | | |
| Cholesterol | Between Groups | 54291.134 | 4 | 13572.783 | 26.891 | .000 |
| | Within Groups | 20189.383 | 40 | 504.735 | | |
| | Total | 74480.517 | 44 | | | |
| Triglycerides | Between Groups | 30859.081 | 4 | 7714.770 | 12.515 | .000 |

| | | | | | | |
|---|---|---|---|---|---|---|
| | Within Groups | 24657.465 | 40 | 616.437 | | |
| | Total | 55516.546 | 44 | | | |

**Table S3:** One-way ANOVA test of tissue antioxidant values and oxidative stress markers.

**ANOVA**

| | | Sum of Squares | df | Mean Square | F | Sig. |
|---|---|---|---|---|---|---|
| G6PDH | Between Groups | 319.892 | 4 | 79.973 | 27.531 | .000 |
| | Within Groups | 116.193 | 40 | 2.905 | | |
| | Total | 436.085 | 44 | | | |
| CAT | Between Groups | 18.835 | 4 | 4.709 | 2.387 | .067 |
| | Within Groups | 78.912 | 40 | 1.973 | | |
| | Total | 97.747 | 44 | | | |
| Antioxidant | Between Groups | 1482.091 | 4 | 370.523 | 18.284 | .000 |
| | Within Groups | 810.610 | 40 | 20.265 | | |
| | Total | 2292.701 | 44 | | | |
| MDA | Between Groups | .322 | 4 | .081 | 83.445 | .000 |
| | Within Groups | .039 | 40 | .001 | | |
| | Total | .361 | 44 | | | |
| SOD | Between Groups | 286.894 | 4 | 71.724 | 62.196 | .000 |
| | Within Groups | 46.127 | 40 | 1.153 | | |
| | Total | 333.022 | 44 | | | |
| GR | Between Groups | 167.193 | 4 | 41.798 | 41.922 | .000 |
| | Within Groups | 39.882 | 40 | .997 | | |
| | Total | 207.075 | 44 | | | |
| GPx | Between Groups | 79.804 | 4 | 19.951 | 113.533 | .000 |
| | Within Groups | 7.029 | 40 | .176 | | |
| | Total | 86.833 | 44 | | | |
